# Effects of enzymatic treatments on the hydrolysis and antigenicity reduction of natural cow milk

**DOI:** 10.1002/fsn3.2066

**Published:** 2020-12-30

**Authors:** Xiaona Liang, Hui Yang, Jing Sun, Jiao Cheng, Xue Luo, Zongzhou Wang, Mei Yang, Dong Bing Tao, Xiqing Yue, Yan Zheng

**Affiliations:** ^1^ College of Food Science Shenyang Agricultural University ShenYang China

**Keywords:** allergic protein, antigenicity, cow milk, enzymatic hydrolysis

## Abstract

Cow milk (CM) allergy is one of the most common food allergies worldwide; the most abundant CM proteins, such as casein (CN), β‐lactoglobulin (β‐LG), and ɑ‐lactalbumin (ɑ‐LA), are all potentially allergenic. Reducing the antigenicity of CM continues to be a major challenge. However, previous studies have focused on the antigenicity of individual allergic CM proteins. Thus, in the present study, we aimed to evaluate the effects of different food‐grade enzymes on the antigenicity of CN, β‐LG, ɑ‐LA in natural CM. The degree of hydrolysis (DH) and molecular mass (MW) distribution of CM hydrolysates were assessed. Additionally, the residual antigenicity of CM hydrolysates was evaluated through enzyme‐linked immunosorbent assay and Western blotting with anti‐CN, anti‐β‐LG, and anti‐ɑ‐LA rabbit polyclonal antibodies. The results showed that Alcalase‐ and Protamex‐mediated hydrolysis could efficiently reduce the antigenicity of CN, β‐LG, and ɑ‐LA, inducing a higher DH, the loss of density of CM proteins, and the increasing levels of low MW (<3 kDa) peptides in CM hydrolysates. Further, Protamex and Alcalase could more efficiently hydrolyze the major allergenic components of CM than the other enzymes, which could represent an advantage for the development of hypoallergenic CM. These findings add further knowledge about the study and development of hypoallergenic CM.

## Graphical abstract

Antigenicity of cow milk proteins can be reduced through enzymatic hydrolysis. Alcalase, Protamex, and Flavourzyme exhibited pronounced hydrolytic activity in cow milk. Alcalase and Protamex decreased the antigenicity of casein and whey proteins significantly.

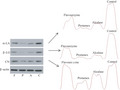

## 1 INTRODUCTION

The incidence of food‐induced allergies and related symptoms continues to increase worldwide. Epidemiological studies have revealed that cow milk (CM) allergy is one of the eight most common food allergies (Wal, 2010); affecting approximately 2%–7.5% of the population in different countries (Pourpak et al., 2004). In addition, CM is one of the most common trigger foods causing food allergy within the first years of life, with 1%–2% of new‐borns exhibiting allergenic responses to CM (Svenning et al., 2000). The immunological reaction can lead to respiratory, dermatological, and gastrointestinal problems, including urticaria, atopic dermatitis, allergic rhinitis, and angioedema (Fiocchi et al., 2010). These symptoms can range from mild to severe anaphylactic reactions, which seriously impact the lives of allergic individuals and the growth of infants and children (Orcajo et al., 2019).

In some parts of the world, CM is one of the most important dietary components for humans and is considered a rich source of proteins, lipids, lactose, and minerals (Liang et al., 2018). Because of its high nutritional value, diverse mineral composition, proper calcium to phosphorus ratio, and numerous sources, it is regarded as the best choice for infant formula and dairy products. However, allergy caused by CM is still a major concern in the dairy industry (Cao et al., 2018).

Previous research has revealed approximately 30 potentially allergenic proteins in CM that can induce immune responses in infants and young children. Moreover, large scale studies on allergic patients have suggested that the most abundant proteins in CM, such as caseins (CN, 78%), β‐lactoglobulin (β‐LG, 9.7%), and ɑ‐lactalbumin (ɑ‐LA, 3.6%), are all major allergens (Aliaga et al., 2020). Even proteins present in low amounts, such as bovine serum (BSA), lactoferrin (LF), and immunoglobulins (Igs), have shown the capacity to induce milk‐related allergies (Kawamoto et al., 2020). Several studies have estimated the incidence of CM protein allergy as approximately 2%–6% (Piippo et al., 2020). Therefore, reducing the antigenicity of CM is a major challenge by milk manufacturers.

Researchers have developed a variety of methods to reduce sensitization to CN, β‐LG, and ɑ‐LA, or other milk components, by optimizing and improving the CM processing conditions (Bu et al., 2013), which including heat treatment, glycation, and enzymatic hydrolysis, and others. Among these, heat treatment is the most commonly used method to prevent pathogens contamination, but it remains a controversial method for reducing the risk of allergies (Fiocchi et al., 2004). Glycation is one of the most frequent chemical modifications during industrial production and processing of milk, but due to the complexity of the product, its safety still warrants evaluation (Taheri‐Kafrani et al., 2009). However, enzymatic hydrolysis, which uses digestive enzymes to alter the immunoreactivity of allergenic proteins, is the most effective method to modify proteins (Wróblewska et al., 2004). During enzymatic hydrolysis, some peptide or disulfide bonds are damaged, leading to the collapse of conformational or linear epitopes; thus, it can eliminate or reduce the allergenicity and antigenicity of milk proteins. Furthermore, enzymatic hydrolysis can yield a variety of new peptides, preserve the nutritional value of milk proteins, and also offer many physiological benefits for infants and young children (Fiocchi et al., 2018).

Enzymatic hydrolysis is a well‐known, safe, and effective processing technology to reduce the allergenicity of milk proteins. Recently, it has been widely used to produce high quality and hypoallergenic protein hydrolysates. Several studies have shown that whey (which contains β‐LG and ɑ‐LA) antigenicity could be reduced by hydrolysis with Alcalase (Docena et al., 2002). Additionally, combined microwave and enzymatic hydrolysis of a whey protein concentrate (WPC) hydrolysate by Pronase, Chymotrypsin, and other five different food‐grade enzymes demonstrated effective proteolysis of whey proteins by either of the enzymes in combination with these treatments (Izquierdo et al., 2006). Some researchers have evaluated the antigenicity of whey proteins hydrolysates obtained through the combination of enzymatic hydrolysis with high‐pressure treatment, suggesting that this method could decrease the immunoreactivity of whey protein hydrolysates (Penas et al., 2006). However, previous studies have focused on the enzymatic hydrolysis of individual proteins or the antigenicity of individual allergic CM proteins. Thus, in the present study, the effects of different food‐grade enzymes on the major allergenic proteins present in natural CM were assessed. Briefly, degree of hydrolysis (DH), molecular weight (MW) distribution, and residual antigenicity of CM hydrolysates were evaluated. This study aimed to lay a solid theoretical foundation for the production of CM‐based hypoallergenic dairy products.

## 2 MATERIAL AND METHODS

### 2.1 Materials

Casein (purity > 85%), ɑ‐LA (purity > 85%), β‐LG (purity > 85%), alkaline phosphatase‐conjugated goat anti‐rabbit IgG antibodies, Freund's complete and incomplete adjuvant, o‐phthalaldehyde (OPA), dithiothreitol (DTT), o‐phenylenediamine dihydrochloride (OPD), Papain and Pepsin were purchased from Sigma‐Aldrich. Alcalase, Neutrase, Protamex, and Flavourzyme were purchased from Novozymes. Three types of rabbit serum comprising polyclonal antibodies targeting CN, ɑ‐LA, β‐LG were prepared at the Shenyang Agricultural University.

### 2.2 Sample collection

Fresh CM was collected at the local farm (Shenyang, China) from 60 healthy cows (1–6 years of age) fed on grass. The CM was mainly composed of protein (3.05 g/100 ml), fat (3.54 g/100 ml), ash (1.12 g/100 ml), moisture (87.80 g/100 ml), lactose (4.98 g/100 ml), and dry matter (12.35 g/100 ml). Fat was removed from the milk by high speed centrifugation at 15,000 g for 30 min at 4°C. Skim milk was placed into bottles, transported to the laboratory in the Shenyang Agricultural University, and stored at 4°C.

### 2.3 Cow milk hydrolysis experiments

The enzyme solutions were prepared by dissolving each enzyme in distilled water (100 mg/ml) at room temperature. The CM samples and enzyme solutions were preheated separately with stirring (20 min) at suitable temperature (Alcalase: 55 ± 5°C; Neutrase: 50 ± 5°C; Flavourzyme: 50 ± 5°C; Protamex: 50 ± 5°C; Papain: 20 ± 5°C; and Pepsin: 30 ± 5°C). Then, each enzyme was added to the CM in an enzyme activity‐to‐substrate ratio ranging between 2,000 and 10,000 U/g. The mixture was incubated for 120 min at their suitable temperature, and the enzymatic hydrolysis reaction was stopped by heating the mixture at 90°C for 10 min, followed by immediate cooling in ice. Afterward, the supernatant of hydrolysates was centrifuged at 5,000 g for 10 min at 4°C and stored at − 80°C for further study. Untreated samples were used as control.

### 2.4 Determination of the DH

The DH of the hydrolysates was evaluated using the OPA method, as previously described (Church et al., 1983), with some modifications. Briefly, the method quantified the amount of hydrolyzed peptide bonds using OPA. The OPA solution was prepared by dissolving sodium dodecyl sulfate (SDS), sodium tetraborate decahydrate, 97% OPA, and 99% DTT. A solution of serine (100 μg/ml) was used as standard control. The OPA reagent (400 μl) was added to the hydrolysates (3 ml), swirled by inversion, incubated for 2 min in the dark, and the absorbance was measured at 340 nm. Each hydrolysate was analyzed in triplicate.

### 2.5 Determination of the molecular weight (MW) distribution

The MW distribution of the CM hydrolysates was assessed by gel permeation chromatography (GPC) using an Agilent PL aquagel‐OH 10 × 300 mm column (LC1260; Agilent) with UV detection at 214 nm under the following conditions: 100 μl injection volume, 30 min analysis time, 30°C column temperature, 0.1 mol/L sodium nitrate, and 500 mg/L sodium azide aqueous solution mobile phase. The column was calibrated using six types of protein as standards: BSA (66.3 kDa), egg ovalbumin (44.5 kDa), soybean trypsin inhibitor (21.5 kDa), cytochrome C (12.3 kDa), pancreas aprotinin (6.5 kDa), Vitamin B_12_ (1.3 kDa), which were all purchased from Sigma‐Aldrich.

### 2.6 SDS‐polyacrylamide gel electrophoresis (PAGE) and Western blotting

Sodium dodecyl sulfate‐polyacrylamide gel electrophoresis was used to evaluate the hydrolysis of CN and whey (β‐LG, ɑ‐LA) in CM hydrolysates, depending on the method described by Laemmli (1970). The separating and stacking gels were prepared by using 15% and 3% of acrylamide concentration, respectively. Prior to electrophoresis, the CM hydrolysates plus loading buffer were heated in boiling water for 5 min. Approximately 15 μg of the protein samples was transferred into each well and the total proteins in the gel were stained with Coomassie Brilliant Blue G‐250.

Next, the gels with the separated proteins were submitted to electroblotting using a miniVE blotter (Bio‐Rad) at 80 V for 120 min, and the proteins were transferred onto a 0.45 μm nitrocellulose membrane (Bio‐Rad). The membrane was incubated with 10 ml blocking buffer (1% BSA (w/v) in phosphate‐buffered saline solution (PBS, pH 8.0) with 0.1% Tween20). Afterward, it was incubated with polyclonal primary antibodies (1:1,000) for 1 hr at 37°C, washed and incubated again with secondary alkaline phosphatase‐conjugated goat anti‐rabbit IgG antibodies (1:5,000 in blocking buffer) for 1 hr at room temperature. Image analysis of the membranes and the proteins was performed with a gel scanner (Amersham Pharmacia Biotech) and the Gel‐pro Analyzer software (Media Cybernetics).

### 2.7 Determination of IgG‐binding ability

The IgG‐binding of CM hydrolysate was quantitatively analyzed by indirect competitive enzyme‐linked immunosorbent assay (ELISA), according to the method by Huang et al. (2019), with some modifications. Microtiter plates (96‐well) were coated with 30 µg/ml CN (or 5 µg/ml ɑ‐LA, or 0.5 µg/ml β‐LG) diluted in PBS and incubated overnight at 4°C. The next day, the plates were washed thrice with 300 µl PBS with 0.05% Tween20 (PBST). Then, 0.5% gelatin in PBS was used to block residual‐free binding sites and the plates were maintained for 1 hr at 37°C. Subsequently, the blocking solution was removed and the plates were washed with PBST. The anti‐proteins that reacted with the plate‐bound antigens were incubated with alkaline phosphatase‐conjugated goat anti‐rabbit IgG antibodies (100 μl; 1:5,000 in PBS) for 1 hr at 37°C. The wells were washed again, followed by the addition of 100 μl of OPD and incubated for 15 min at 37°C in the dark. After 15 min, the reaction was stopped by adding 50 μl of 2 M H_2_SO_4_. The absorbance was measured at 490 nm by using an Eon microplate spectrophotometer (Biotek Instruments Inc., Winooski, VT, USA). The IgG reactivity inhibition (%) was calculated based on the following equation:

$$\text{IgG}\;\text{reactivity}\;\text{inhibition}\;\left(\%\right)=\left(1‐\frac{B}{B_{0}}\right)*100$$

where *B* and *B*_0_ represent the absorbance measured in the presence and absence of CM hydrolysate, respectively.

### 2.8 Statistical analysis

The results are expressed as the mean ± standard deviation of three independent assays. Analysis of variance (ANOVA) was used to assess the effects of the treatments and differences between samples. The analyses were performed using SPSS Statistics for Windows software version 17.0 (SPSS, Inc.). Differences were considered significant at *p* < .05.

## 3 RESULTS AND DISCUSSION

### 3.1 The degree of hydrolysis

**Figure 1:**
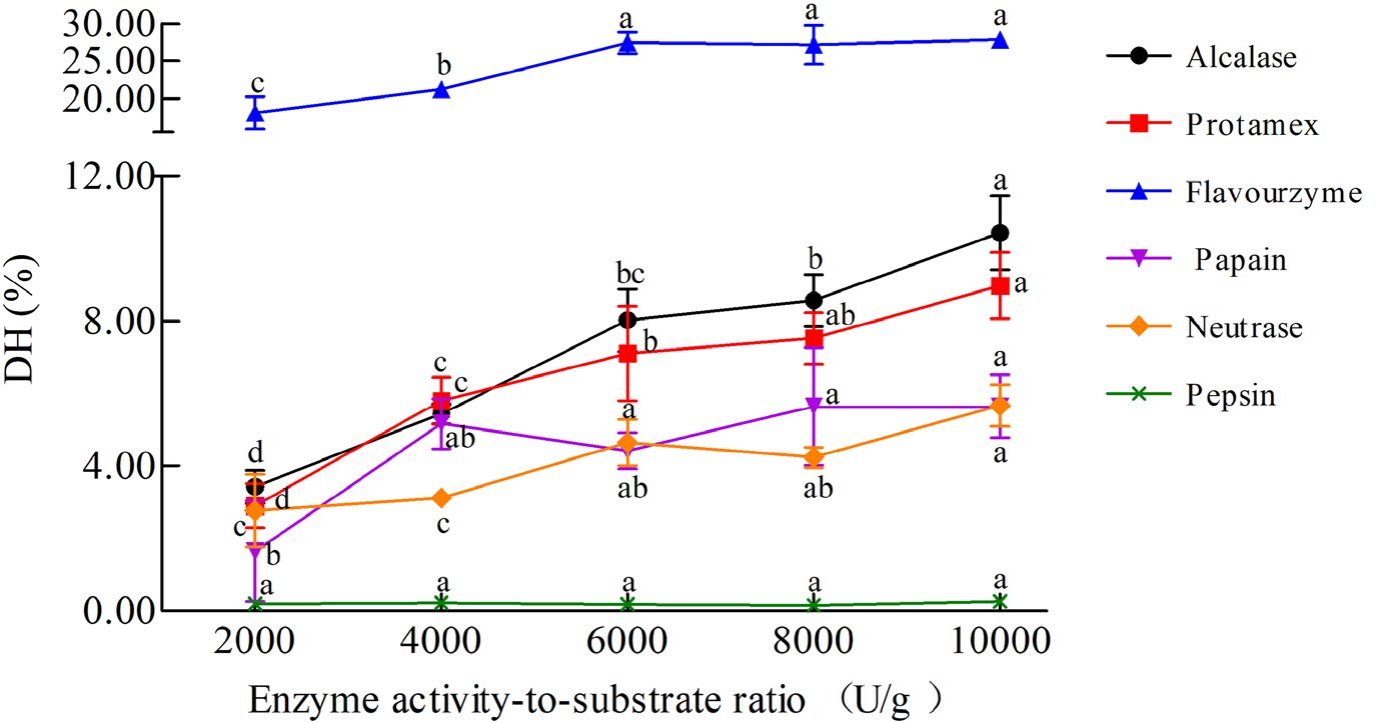
The degree of hydrolysis (DH) of cow milk with different enzyme activity‐to‐substrate ratio (U/g). Each value represents the mean of three independent experiments ± *SD*. Different letters indicate significant differences among groups (*p* < .05)

The DH of CM hydrolysates obtained with different enzymes was quantified using the OPA method, as shown in Figure 1. The DH of CM hydrolysates was observed ranging from 0.16% to 27.80%, possibly due to the different enzyme systems or activities during the enzymatic hydrolysis. The DH of the CM hydrolysates obtained using Pepsin was 0.27%, whereas the CM subjected to Flavourzyme hydrolysis exhibited a higher DH ranging from 18.06% to 27.80%. Moreover, hydrolysis treatment using Flavourzyme, Protamex, and Alcalase was found to be more effective than with the other enzymes. In addition, increased enzyme activity‐to‐substrate ratio of these enzymes was found to be associated with gradual DH increase of the CM hydrolysates, reaching a maximum hydrolysis of 27.80%, 8.98%, 10.44% with Flavourzyme, Protamex, and Alcalase, respectively, when the enzyme activity‐to‐substrate ratio reached 10,000 U/g. It should be noted that these enzymes played an important role in improving the DH of CM; thus, they could be considered superior to the other enzymes.

To date, enzymatic hydrolysis is the most efficient method to decrease allergenicity and antigenicity of CM proteins. The procedure of enzymatic hydrolysis could disrupt conformation or linear epitopes and prevent IgE‐mediated allergic reactions. The enzymatic hydrolysis of milk proteins is a vital step in the development of hypoallergenic milk for allergic infants and children (Oliveira et al., 2019). The degree of milk proteins hydrolysis is influenced by enzyme specificity, types of enzymes, and enzymatic hydrolysis conditions (pH, enzyme to substrate ratio (E/S), hydrolysis time, and temperature) (Cheison et al., 2007). Ena et al. (2010) demonstrated that WPC hydrolysate obtained with Alcalase was characterized by a DH ranging from 14.5% to 18%. Spellman et al. (2003) also found a similar DH of WPC, that is, 14% with Alcalase 2.4 L.

### 3.2 SDS‐PAGE

**Figure 2:**
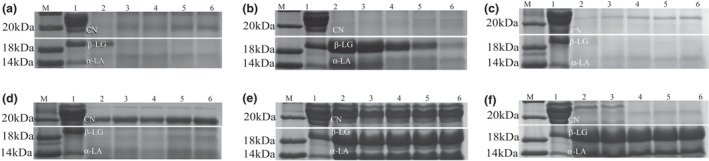
SDS‐PAGE analysis. (a–f) Represent flavourzyme, protamex, alcalase, papain, pepsin, neutrase, respectively. M: marker; lane 1: cow milk; lane 2:2,000 :1; lane 3:4,000 :1; lane 4:6,000 :1; lane 5:8,000 :1; lane 6:10,000:1

The antigenic proteins of CM are mainly made up of CN, β‐LG, ɑ‐LA with MW of 19.0–25.2 kDa, 18.3 kDa, 14.2 kDa, respectively. The protein patterns of CM and hydrolysates (Figure 2) indicated that the electropherogram of the CM proteins changed significantly after the enzymatic hydrolysis. Overall, in comparison with CM, the hydrolysates had fewer larger MW proteins, whereas and lower MW proteins bands increased distinctly. Moreover, the density of the protein bands was also altered. As seen in Figure 2, Alcalase, Protamex, and Flavourzyme showed strong hydrolysis potential for those major allergenic proteins, among which Alcalase and Flavourzyme extensively hydrolyzed CN and whey (β‐LG, ɑ‐LA) even at the lowest concentration assessed (enzyme activity‐to‐substrate ratio 2,000 U/g). However, increased enzyme activity‐to‐substrate ratio had no significant impact on the protein bands. In addition, Protamex was associated with a concentration‐dependent hydrolysis, in which the density of the protein bands gradually reduced with increasing enzyme activity‐to‐substrate ratio. Notably, Pepsin, Papain, and Neutrase were unable to completely hydrolyze CN and whey (β‐LG, ɑ‐LA). Therefore, among all the different enzymes used herein, Alcalase, Protamex, Flavourzyme were superior to the other enzymes in their ability to degrade antigenic proteins of CM.

Enzymatic hydrolysis can breakdown the peptide bonds of milk proteins and convert the whole proteins into smaller peptide fragments. Due to differences in enzymes species, their degradation ability for targeting proteins may be different. Smyth and Fitzgerald (1998) hydrolyzed a WPC preparation using Alcalase 0.6 L. After 30 min of enzymatic hydrolysis, they observed that the presence of a protein fraction of MW below 30 kDa. In addition, Ena et al. (2010) studied the protein conformation changes in WPC preparation during hydrolysis with Pepsin and Corolase PP. In this study, after 30 min of hydrolysis, small fractions containing proteins with MW of over 16 kDa were observed, suggesting different proteolytic enzyme specificities.

### 3.3 IgG reactivity reduction analysis

**Figure 3:**
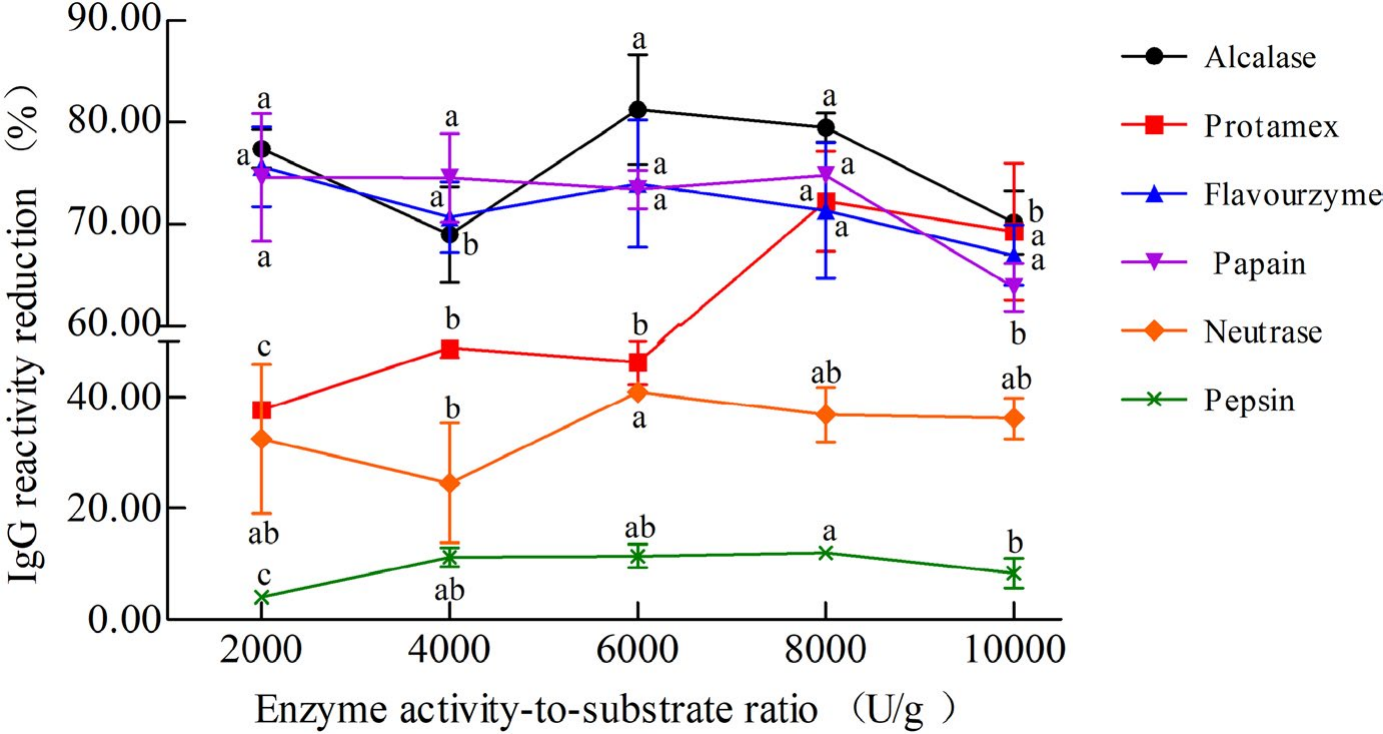
IgG reactivity of cow milk hydrolyzed with different enzymes and enzyme activity‐to‐substrate ratio (U/g). Each value represents the mean of three independent experiments ± *SD*. Different letters indicate significant differences among groups (*p* < .05)

To identify the IgG reactivity reduction of samples obtained by the different enzymes, rabbit polyclonal antibody and indirect competitive ELISA were performed. As shown in Figure 3, the IgG reactivity reduction of CM hydrolysates ranged from 4.02% up to 81.27%. The IgG reactivity reduction of CM hydrolysates obtained with Pepsin was the lowest (4.02%–12.02%). In turn, Protamex‐derived IgG reactivity reduction increased gradually with increasing enzyme activity‐to‐substrate ratio, reaching a maximum of 72.25% when the enzyme activity‐to‐substrate ratio was at 8,000 U/g. In addition, it could be observed that CM subjected to Flavourzyme, Papain, or Alcalase hydrolysis exhibited higher IgG reactivity reduction (69.00%–81.27%), which was in agreements with the DH results (Section 3.1, Figure 1). The IgG reactivity reduction indicated that the CM antigenic epitopes were altered upon the enzymatic hydrolysis, although with slightly variable outcomes. Additionally, it also suggested that enzymatic hydrolysis can change the structure of the allergens in CM, interfere with the antigen‐antibody complex, and thus reduce the IgG reactivity. Some studies have demonstrated that the residual antigenicity and IgE‐binding ability of ɑ‐LA, β‐LG, α‐CN, β‐CN in CM with reduction rate of approximately 15%–90% (Shi et al., 2014).

### 3.4 Molecular weight distribution

**Figure 4:**
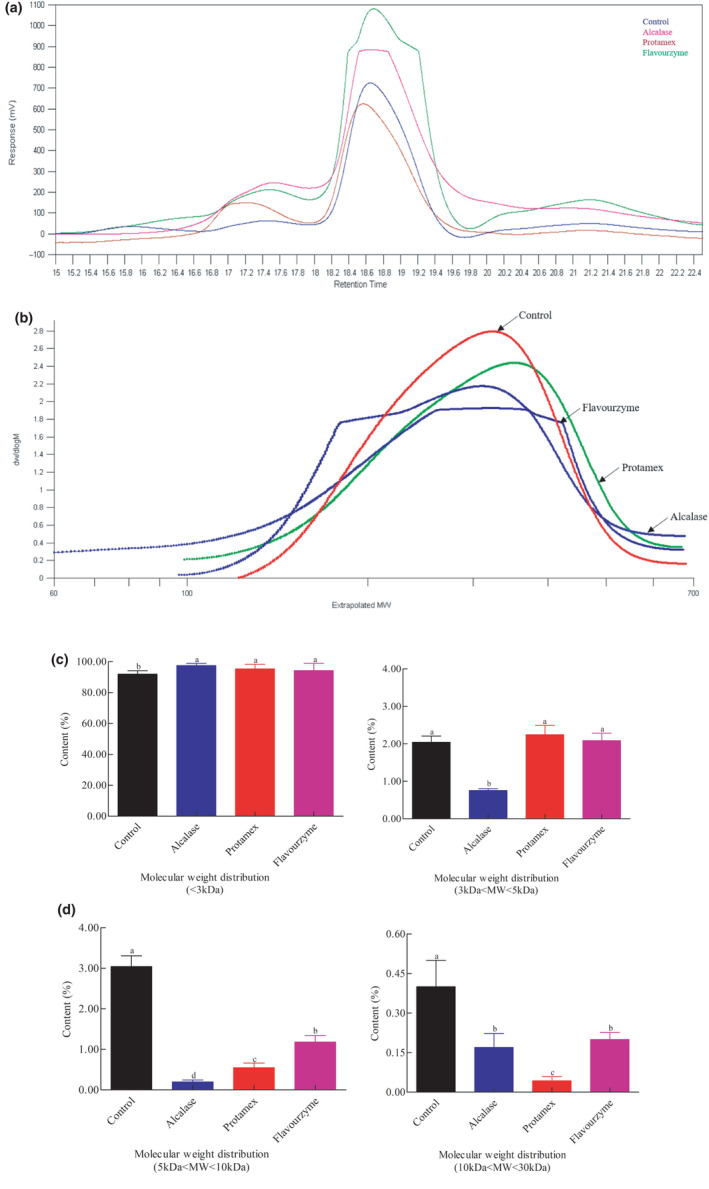
Molecular weight distribution of cow milk hydrolysates determined by gel permeation chromatography. Each value represents the mean of three independent experiments ± *SD*. Different letters indicate significant differences among groups (*p* < .05)

Enzymatic hydrolysis could break down milk proteins into short peptides and change the MW distribution, which was evaluated by GPC. The MW distribution was calculated assuming an exponential relationship between MW and elution time. After enzymatic hydrolysis with different enzymes, the MW distribution of the CM hydrolysates was significantly different, which reflected the differences observed in the peptide chain lengths. The MW distribution of the CM and CM hydrolysates is presented in Figure 4. It was clear that the MW of CM hydrolysates was mostly composed of small peptides (<3 kDa). After enzymatic hydrolysis, the percentage of milk proteins with MW distribution of 10–30 kDa, 5–10 kDa, 3–5 kDa significantly reduced (*p* < .05), suggesting that the cleavage reaction triggered by the different enzymes led to the fragmentation of the CM proteins into low MW peptides. Furthermore, the MW distribution of CM hydrolysates showed considerable differences. For the CM hydrolysate obtained with Flavourzyme, the relative percentage of milk proteins with a MW distribution of 3–5 kDa was approximately 2.05%, whereas for CM hydrolysate treated with Alcalase was of 0.76%. These results in combination with DH and SDS‐PAGE data suggest that enzymatic hydrolysis with Alcalase and Protamex were much more effective in producing smaller peptides from CM. Several studies proved that the most effective strategy to reduce the allergenicity of CM is by decreasing the MW of the principal CM allergens, namely CN, β‐LG, and ɑ‐LA, through enzymatic proteolysis (Asselin et al., 2010). Furthermore, some investigations have shown that peptides with MW between 1.6 and 3.5 KDa, prepared from either CN or whey, were unable to elicit an IgE‐mediated allergic response (Otani et al., 1989). Deeslie and Cheryan (2010) reported that peptide MW was also one of the key factors regulating the functional properties of CM hydrolysates.

### 3.5 Western blotting

**Figure 5:**
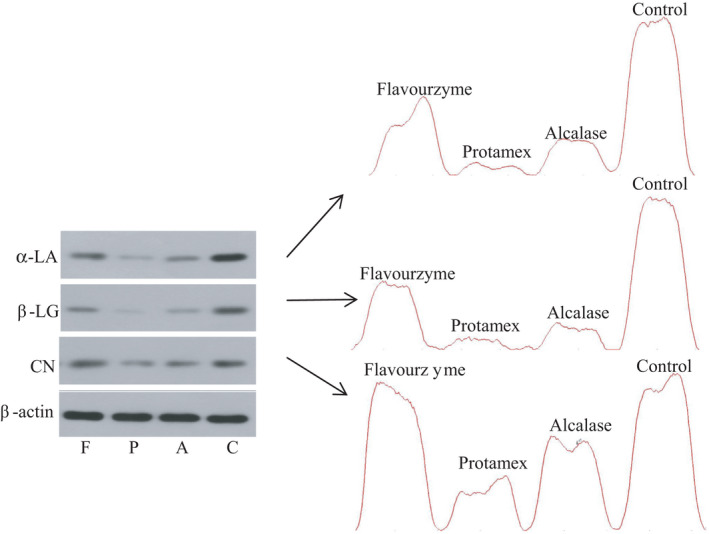
Immunoblot of polypeptides obtained by hydrolysis of cow milk using three enzymes. C: Control (unhydrolyzed cow milk); A: Alcalase; P: Protamex; F: Flavourzyme

To study the changes in allergenicity of the main CM allergenic proteins after enzymatic hydrolysis, Western blotting analysis using anti‐CN, anti‐β‐LG, anti‐ɑ‐LA polyclonal antibodies was performed. The CM proteins before and after the enzymatic hydrolysis had an immune binding reaction with the IgG antibody of the anti‐CN, anti‐β‐LG, and anti‐ɑ‐LA rabbit serum (Figure 5), indicating that the CM proteins before and after the enzymatic hydrolysis were immunoreactive. Compared with CM, the IgG‐binding capacity of the antigen proteins (CN, β‐LG, ɑ‐LA) in CM hydrolysate was significantly reduced; however, the IgG‐binding capacity of CN in CM hydrolysate was stronger than that of β‐LG and ɑ‐LA. Similar results demonstrated that Alcalase was more effective at reducing antigenicity of milk proteins, as it significantly reduced in the IgG‐binding capacity of α‐LA and β‐LG (Yu et al., 2019). Carvalho et al. (2017) reported that Alcalase hydrolysis had similar inhibitory effect on the IgE‐ or IgG‐binding capacity of α‐LA and β‐LG in WPC.

### 3.6 IgG‐binding ability

**Figure 6:**
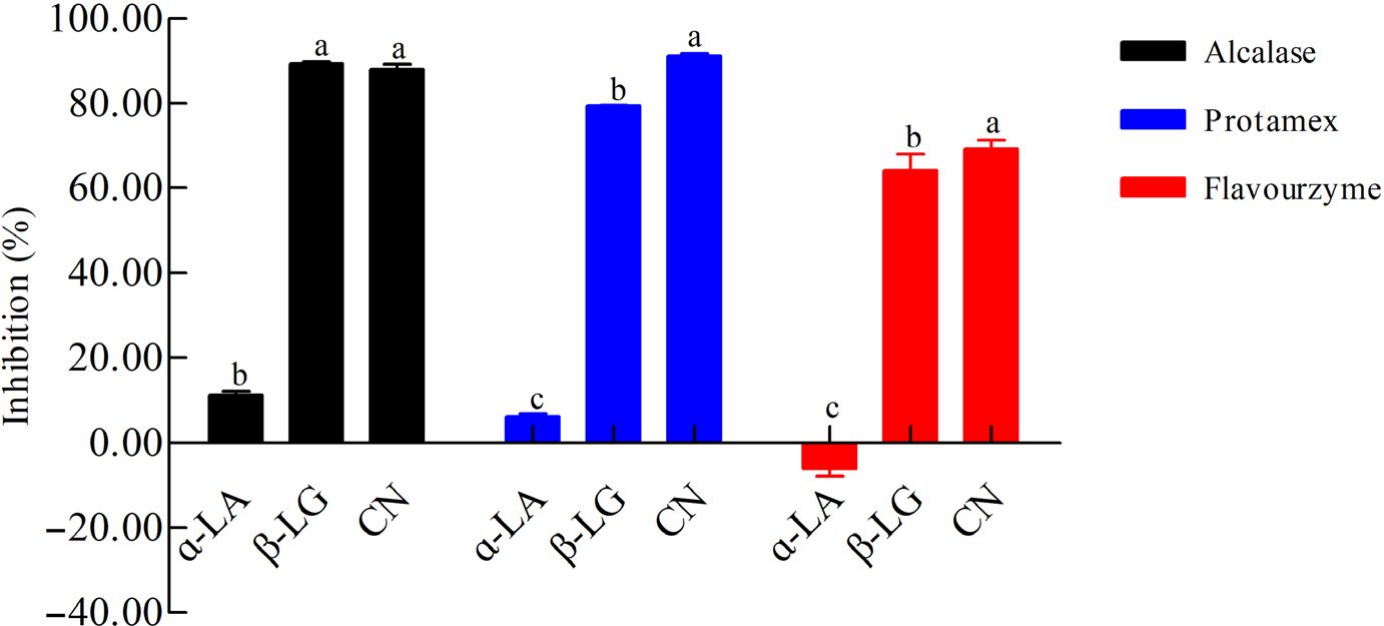
Residual antigenicity of cow milk hydrolysates determined by ELISA using polyclonal antibodies against ɑ‐LA, β‐LG, and CN. Each value represents the mean of three independent experiments ± *SD*. Different letters indicate significant differences among groups (*p* < .05)

To determine the antigenicity of the CM hydrolysates, polyclonal antibodies were prepared, and competitive inhibition ELISA was used to evaluate the residual antigenicity of CN, β‐LG, and ɑ‐LA. Overall, enzymatic hydrolysis could reduce the antigenicity of CM proteins, in which the antigenicity reduction rate of β‐LG and CN was much higher than that of ɑ‐LA (*p* < .05). Furthermore, Alcalase‐ and Protamex‐mediated enzymatic hydrolysis of CM led to an ɑ‐LA IgG‐binding reduction rate of 11.28% and 6.10%, respectively, whereas a significantly higher residual antigenicity was obtained with Flavourzyme (−6.09%) (*p* < .05, Figure 6). In addition, the IgG‐binding ability of β‐LG during enzymatic hydrolysis with Flavourzyme and Protamex was significantly reduced (59.09% and 79.37%, respectively), but the enzymatic hydrolysis with Alcalase led to a dramatic reduction of 90.25% (*p* < .05, Figure 6). A marked decrease in IgG‐binding potential of CN during the enzymatic hydrolysis process could also be observed, with a significant reduction rate of 69.14%–91.21%. It is worth noting that Alcalase and Protamex played an important role in reducing the antigenicity of CN, β‐LG, and ɑ‐LA; therefore, it could be concluded that they were superior to the other enzymes tested. These findings are in agreement with previously reported data. Wróblewska and Troszyñska (2005) showed that the lowest immunoreactivity to anti‐ɑ‐LA antibodies was found for whey protein hydrolysate obtained using Alcalase and its double dose. Quintieri et al. (2017) also reported that the antigenicity of whey was slightly reduced when it was incubated with fungal proteinases and pancreatic extracts.

In the present study, the amount of low MW peptides was significantly increased and the IgG‐binding ability was considerably reduced in CM hydrolysates. This combined effect can be explained by the underlying enzymatic reaction, which partially breakdowns large proteins into low MW peptides, concomitantly, it may also split the sequence of epitopes, thereby resulting in reduced antigenicity. The allergic epitopes could be damaged or destroyed by the degradation of milk proteins or occurring conformational changes, which resulted in reduced reactivity and IgG‐binding ability. In addition, the enzyme specificity or types can also affect the degradation of allergic epitopes. Similar research evaluating the effects of 12 different food‐grade enzymes also revealed that WPC incubated with Papain had the lowest IgE‐binding ability (Biela et al., 2019). Moreover, Villas‐Boas et al. (2015) demonstrated that enzymatic hydrolysis could reduce the number of allergic epitopes, and induce marked reduction of the IgE‐ or IgG‐binding ability of β‐LG.

## 4 CONCLUSIONS

In this study, natural CM was first subjected to enzymatic hydrolysis by different food‐grade enzymes, and the effects on the major allergenic proteins were assessed. The results showed that Alcalase and Protamex hydrolysis could efficiently reduce the antigenicity of CM allergenic proteins, in particular of CN and β‐LG, while showing a higher DH and the loss of density of CM proteins. In addition, hydrolysis treatment led to a marked increase of low MW (<3 kDa) peptide in CM hydrolysates. Overall, Protamex and Alcalase more efficiently hydrolyzed the major allergenic compounds of CM than the other enzymes, representing potential tools for the development of hypoallergenic CM. This study adds further knowledge to the field, as previous studies have been limited to the enzymatic hydrolysis of individual proteins or the antigenicity of individual allergic proteins of CM. In conclusion, this study provides experimental and theoretical evidence for further research on hypoallergenic CM.

## CONFLICTS OF INTEREST

The authors confirm that the contents of this article pose no conflicts of interest.

## Data Availability

Data available on request from the authors—The data that support the findings of this study are available from the corresponding author upon reasonable request.
